# Photoacclimation strategies in cyanobacterial photosynthesis under dynamic light environments: implications in growth, fitness, and biotechnological applications

**DOI:** 10.3389/fmicb.2025.1686386

**Published:** 2025-10-20

**Authors:** Sapna Tiwari, Anjali Gupta, Deepa Pandey, Priyul Pandey, Rinkesh Gupta, Shailendra Pratap Singh

**Affiliations:** Department of Botany, Institute of Science, Banaras Hindu University, Varanasi, UP, India

**Keywords:** biomanufacturing, cyanobacteriochrome, non-photochemical quenching, photoprotection, synthetic biology

## Abstract

Cyanobacteria, ancient oxygenic photoautotrophs originated in the Precambrian period, exhibit remarkable adaptability to diverse ecological systems. Light, a critical environmental factor, exerts differential pressures on these organisms. The scattering of white light creates dynamic light environments, which poses a significant ecological challenge. To thrive in dynamic light environment, cyanobacteria have developed several light acclimation strategies. This includes chromatic acclimation, which optimize light harvesting by adjusting pigments. Cyanobacteria also employ robust photoprotective mechanisms against quantitative light stress. Under high light, these organisms activate non-photochemical quenching using the proteins such as orange carotenoid protein, iron starvation-induced protein, and high light-induced proteins to safely dissipate excess excitation energy. Additionally, thylakoid-localized respiratory enzymes alleviate electronic pressure arising from over-reduction of the plastoquinone pool. Under low light conditions, cyanobacteria frequently employ state transitions, reversibly associating their phycobilisomes with PSII and PSI to optimize light harvesting. These natural strategies offer a blueprint for engineering cyanobacteria and algae for their application in biomanufacturing and CO_2_ sequestration. This review synthesizes the key light acclimation and photoprotective mechanisms, underscoring their importance for both the ecological success of cyanobacteria and their implication in biotechnological applications using engineered strains.

## Introduction

The groundbreaking oxygen production by cyanobacteria triggered the evolution of aerobic life. The subsequent rise in oxygen levels, a byproduct of their photosynthesis, further catalyzed the formation of the stratospheric ozone layer, which shields Earth from lethal UV-C (100–280 nm) radiation (Sánchez-Baracaldo et al., 2022). Thus, early cyanobacterial oxygenic photosynthesis not only drove the emergence of aerobic organisms but also established a crucial protective ozone shield. While many cyanobacteria are free-living in diverse aquatic and terrestrial environments, they also form symbiotic relationships with lichens, bryophytes, and gymnosperms (Álvarez et al., 2023). Notably, the endosymbiotic integration of cyanobacteria into non-photosynthetic eukaryotes ultimately led to the evolution of chloroplasts (Zimorski et al., 2014).

Cyanobacteria boast remarkable morphological and metabolic diversity, enabling their survival across varied ecological niches. Within these environments, they play crucial roles in food webs and the fixation of both dinitrogen and carbon dioxide (CO_2_) (Garcia-Pichel and Belnap, 2021). Furthermore, cyanobacteria can be metabolically engineered to redirect fixed carbon and nitrogen into the production of valuable chemicals, including biofuels, bioplastics, and diverse metabolites (Baunach et al., 2024; Rueda et al., 2024; Singh et al., 2017). Nevertheless, the effective use of cyanobacteria for large-scale production of energy-rich molecules and valuable chemicals, as well as their application in CO_2_ capturing or agriculture as biofertilizers for achieving the United Nations’ sustainable development goals, hinges on fundamental photosynthetic function (Pandey et al., 2025). Cyanobacterial growth, whether in natural settings or large-scale cultivation systems, is susceptible to light fluctuations. These fluctuations encompass diurnal, seasonal, and spatial variations in light quality and quantity, leading to imbalanced photosystem excitation that compromises photochemistry (Maurya et al., 2023; Pfennig et al., 2024; Canonico et al., 2021). Besides suboptimal levels of photosynthetically active radiation (PAR; 400–700 nm), ultraviolet radiation (UVR; 280–400 nm) can directly damage the photosynthetic apparatus or indirectly by inducing the production of reactive oxygen species (ROS) (Kataria et al., 2014). However, cyanobacteria have evolved diverse acclimatization strategies to modulate their development and photosynthetic machinery in response to light signals (Gupta et al., 2023; Konert et al., 2023).

**Figure 1:**
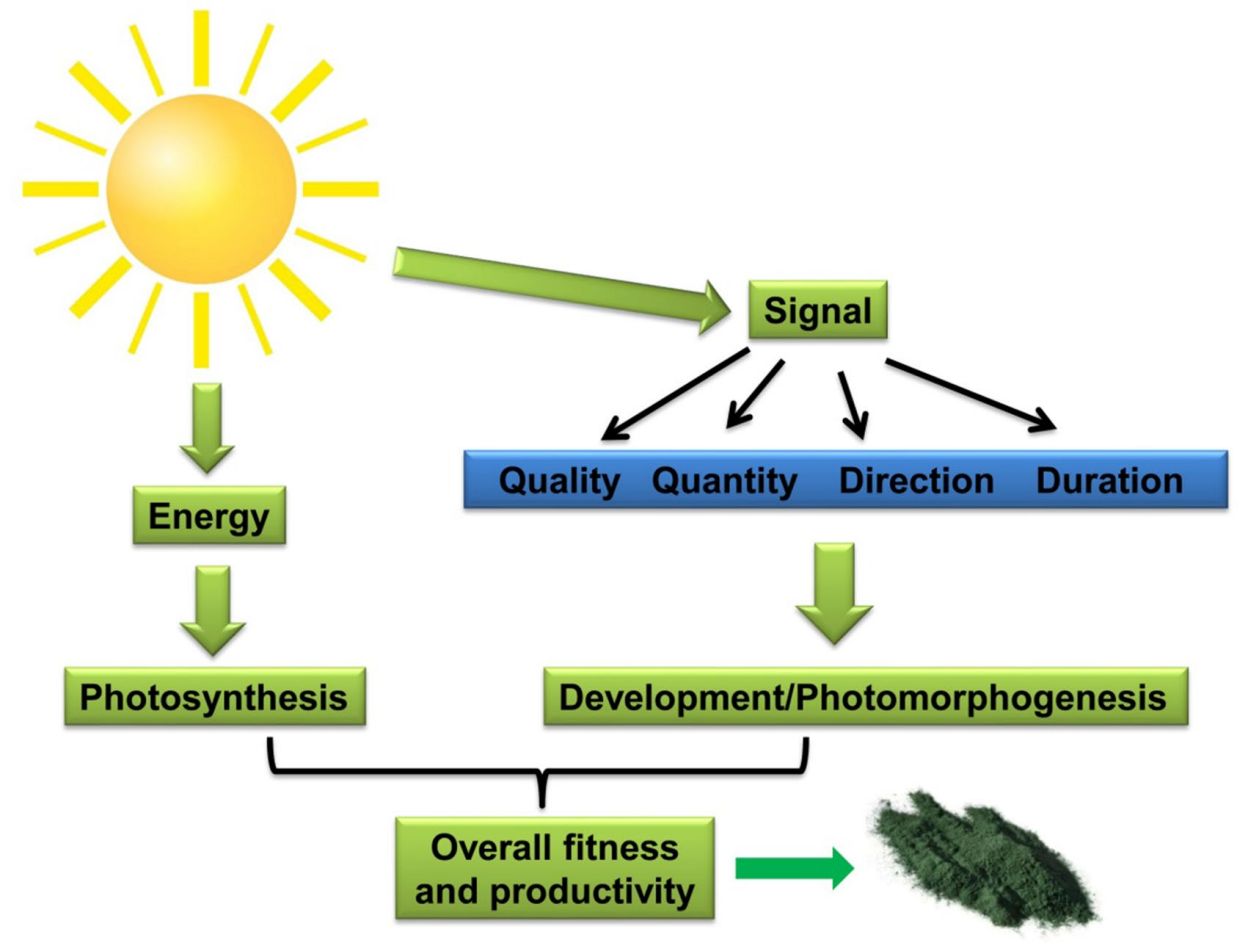
Light energy exerts a dual influence on photoautotrophs, driving both photosynthesis and acting as a signal for developmental processes (photomorphogenesis). The balance between energy gained through photosynthesis and its allocation towards developmental repair and maintenance ultimately determines the organism’s fitness, reflected in biomass accumulation.

Therefore, solar radiation serves as the primary energy source for photochemistry in all photoautotrophs, including cyanobacteria. Its quality, quantity, direction, and duration also act as key environmental cues that drive developmental and/or morphogenetic programs (Figure 1). This light-driven developmental regulation, termed photomorphogenesis, optimizes organismal fitness by enhancing growth and photon resource utilization (Mondal et al., 2024). Ultimately, biomass production reflects a balance between photosynthetic energy capture and its allocation towards developmental programs and other cellular processes, such as repair mechanisms that promote survival and fitness (Figure 1). Owing to their extensive phenotypic and genotypic diversity, cyanobacteria exhibit a wide array of photoacclimatization strategies. These strategies not only safeguard photosystem (PS) function but also enable efficient photosynthesis under varying light conditions. Notably, these acclimation strategies could significantly improve the biomass production in the large-scale cultivation system. Therefore, these strategies are ideal characteristics for engineering cyanobacteria to enhance photosynthetic performance in fluctuating light conditions (Silkina et al., 2019; Battaglino et al., 2021).

Under high light stress, cyanobacteria employ diverse strategies to manage both physical and chemical excitation pressure on photosystem II (PSII). Physical balancing of light energy is achieved through mechanisms involving the orange carotenoid protein (OCP) (Domínguez-Martín et al., 2022), iron-starvation inducible protein (IsiA) (Nagao et al., 2023), high-light inducible proteins (HliPs) (Konert et al., 2023), and state transitions (Calzadilla and Kirilovsky, 2020). The electronic load on PSII is dissipated by respiratory electron transport chain complexes and flavodiiron proteins (Ermakova et al., 2016; He et al., 2025). This interconnected electron transport network helps to alleviate electronic pressure on both PSII and PSI in cyanobacteria. However, when these initial defense systems are overwhelmed by excessive light, various antioxidative systems are activated to shield cells from oxidative damage caused by ROS (Kataria et al., 2014). In addition to protecting PSII, cyanobacteria also exhibit photophobic or phototactic movements, enabling them to move away from or towards light sources, respectively (Gupta et al., 2023).

Light quality also presents a significant ecological challenge, and consequently, cyanobacteria restructure their phycobilisomes (PBSs) by altering phycobiliproteins (PBPs), linker proteins, or chlorophylls (Chl) and carotenoids (Cars). These changes, triggered by light signals, optimize the absorption of available photons for efficient photosynthesis in fluctuating light environments (Gupta et al., 2023). These developmental responses to light color or intensity are termed chromatic acclimation (CA). CA provides an additional advantage to cyanobacteria by modulating their capacity to absorb the prevailing wavelengths of light (Mondal et al., 2024).

This review synthesizes the diverse cellular damages inflicted by high light and UVR stress and comprehensively accounts for the strategies employed by cyanobacteria under varying light conditions. We highlight the superior flexibility and diversity of cyanobacteria compared to plants in tolerating light stress, positioning them as highly efficient photoautotrophs for utilizing natural light. Ultimately, understanding the intricate photoprotective and photoacclimatory mechanisms evolved by these ancient and metabolically versatile prokaryotes—including energy dissipation, antioxidative responses, and CA—is crucial. This knowledge not only elucidates their ecological success and significant role in shaping Earth’s environment but also paves the way for effectively harnessing their remarkable oxygenic photosynthesis for sustainable biotechnological applications to achieve United Nations’ sustainable developmental goals.

## Cyanobacterial thylakoid membranes: structure and evolutionary significance

Cyanobacteria, Gram-negative photosynthetic bacteria, exhibit an ultrastructure similar to plant chloroplasts. Their photosynthetic machinery resides in thylakoid membranes (TMs), which are typically arranged as concentric rings towards the periphery of the cytoplasm, enclosing a lumen (Allen and Forsberg, 2001). However, TM distribution can vary with environmental conditions (Huokko et al., 2021). The structural similarities and phylogenetic evidence support the endosymbiotic theory of chloroplast origin from cyanobacteria (Stadnichuk and Kusnetsov, 2021; Whatley et al., 1979).

**Figure 2:**
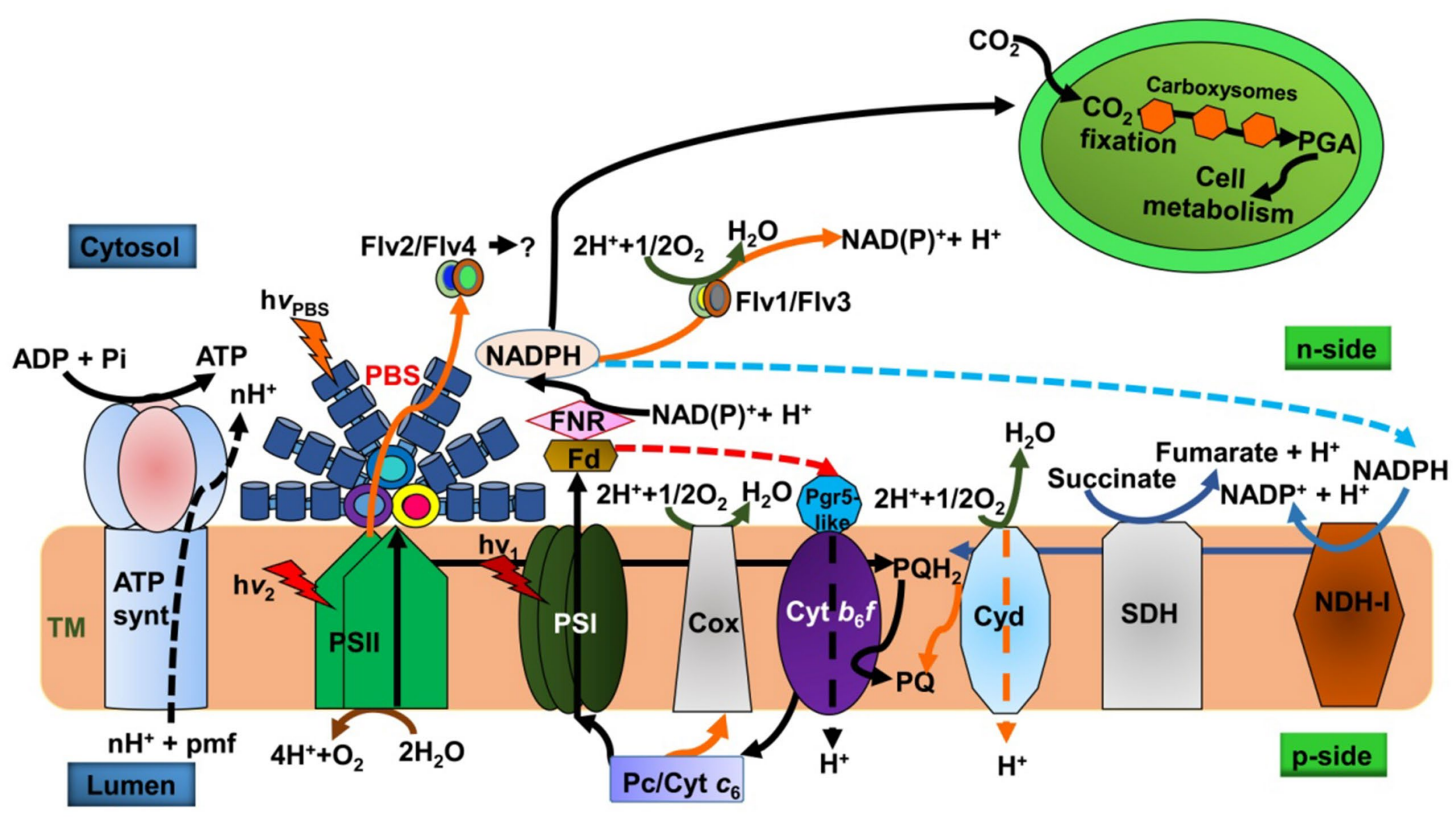
Schematic diagram of photosynthetic and respiratory electron transport on the thylakoid membrane (TM). The Z-scheme (black arrows) depicts light absorption by PBSs exciting PSII, followed by water splitting (brown arrow) in the lumen. Cyclic electron transport (red dashed arrow) around PSI generates ATP. Respiratory complexes SDH and NDH-I (blue arrows) reduce the PQ-pool; SDH oxidizes succinate to fumarate, while NDH-I oxidizes NADPH (blue dashed arrow). Orange arrows indicate PSII protection mechanisms involving flavodiiron proteins Flv2/Flv4 (associated with PSII) and Flv1/Flv3 (associated with PSI), which alleviate electronic stress. Complexes Cox, Cyd, and Flv1/Flv3 release water (dark green arrow) by accepting electrons from Pc, PQH2, and NADPH, respectively (Stirbet et al., 2019; Liu, 2016). TM, thylakoid membrane; PSII, photosystem II; NDH-I, NADPH-quinone dehydrogenase; SDH, succinate dehydrogenase; hν_PBS_, light absorption by phycobilisomes (PBS); h*ν*_1_, light absorption by PSI; h*ν*_2_, light absorption by PSII; Flv1/3 and Flv2/4, flavodiiron protein; PQ, plastoquinone; Cyd, cytochrome *bd* quinol oxidase; Cyt *b*_6_*f*, cytochrome *b*_6_*f*; Pc, plastocyanin; Cyt *c*_6_, cytochrome *c*_6_; NAD(P)H, nicotinamide-adenine dinucleotide phosphate; Fd, ferredoxin; FNR, ferredoxin-NADP^+^ oxidoreductase; Cox, cytochrome *c* oxidase; Pgr5, proton gradient regulation; H^+^, proton; ATP syn, adenosine triphosphate synthase; pmf, proton motive force; p-side, lumen side; n-side, cytosolic side.

The endosymbiotic event likely drove TM folding in chloroplast ancestors to accommodate cyanobacteria-like endosymbionts within the host cell’s limited space. Furthermore, the loss of PBSs and an increased PSII cross-section facilitated thylakoid stacking and the spatial segregation of PSII (in grana lamellae) and PSI (in stroma lamellae), minimizing excitation energy spillover. However, far-red light, preferentially absorbed by PSI, can induce PSI migration to grana lamellae, potentially causing spillover (Terashima et al., 2025; Stadnichuk and Kusnetsov, 2021). Thus, endosymbiotic membrane folding resulted in the stacked (grana lamellae) and unstacked (stromal lamellae) thylakoids characteristic of modern plant chloroplasts, contrasting with the appressed thylakoids in cyanobacteria (Huokko et al., 2021). The close homology between cyanobacteria and chloroplasts offers a valuable system to investigate photosynthetic mechanisms and responses to environmental factors, aiding our understanding of photophysiology across cyanobacteria, algae, and plants. Cyanobacteria also serve as a model for studying chloroplast evolution and biogenesis (Nielsen et al., 2016). Notably, cyanobacterial thylakoids house electron carriers for both photosynthesis and respiration (Figure 2). These processes are interconnected in cyanobacteria (Mullineaux, 2014), which is unlike the compartmentalized photosynthesis (in chloroplast) and respiration (in mitochondria) found in algae and higher plants.

In *Synechocystis* sp. PCC 6803, PSII resides in the outer TM, while PSI is located in the inner TM (Liu, 2016). Conversely, in *Synechococcus elongatus* PCC 7942, PSI is peripheral, and PSII is uniformly distributed within the TM (Liu, 2016). Cyanobacterial PSII exhibits a granular structure due to the attachment of its core light-harvesting antenna, PBSs—unlike the peripheral antennae in plants—on the TM’s outer surface. This PBS attachment gives a granular appearance between TMs. Unlike plants, cyanobacteria lack thylakoid stacking; instead, their photosynthetic membranes display diverse peripheral distribution patterns (Mareš et al., 2019). The presence of PBSs prevents membrane appression, but their absence in mutants or specific light conditions can induce thylakoid appression in cyanobacteria (Maurya et al., 2023; Liberton et al., 2013). The branched TMs have been reported in *Cyanothece* sp. 51,142 where photosynthetic membranes are interconnected through channels and share a common lumen (Liberton et al., 2011).

PBSs function as light-harvesting antennae, analogous to those in higher plants, increasing the absorption cross-section for PSII photochemistry. Cryogenic confocal microscopy indicates that PBSs primarily transfer excitation energy to PSII but can also directly interact with PSI or form PBS-PSII-PSI supercomplexes (Steinbach et al., 2015). The delicate TM, crucial for cellular physiological stability, is susceptible to environmental influences. Light and other factors can alter TM lipid fluidity, leading to topographical changes (Mullineaux and Liu, 2020). While cryoelectron microscopy has been used to study PS topography, its freeze-fracture method can compromise the native structure. Atomic force microscopy (AFM) is emerging as an alternative for studying TM topography in its physiologically active state within aqueous media. AFM offers valuable insights into PS topography, revealing that PSII and PSI can dynamically change their structure in response to environmental conditions (MacGregor-Chatwin et al., 2019; Zhao et al., 2022). Consequently, AFM is a promising tool for exploring the structural and mechanical aspects of photosynthesis that remain largely unknown.

## Structural adaptations in photosynthetic machine

**Figure 3:**
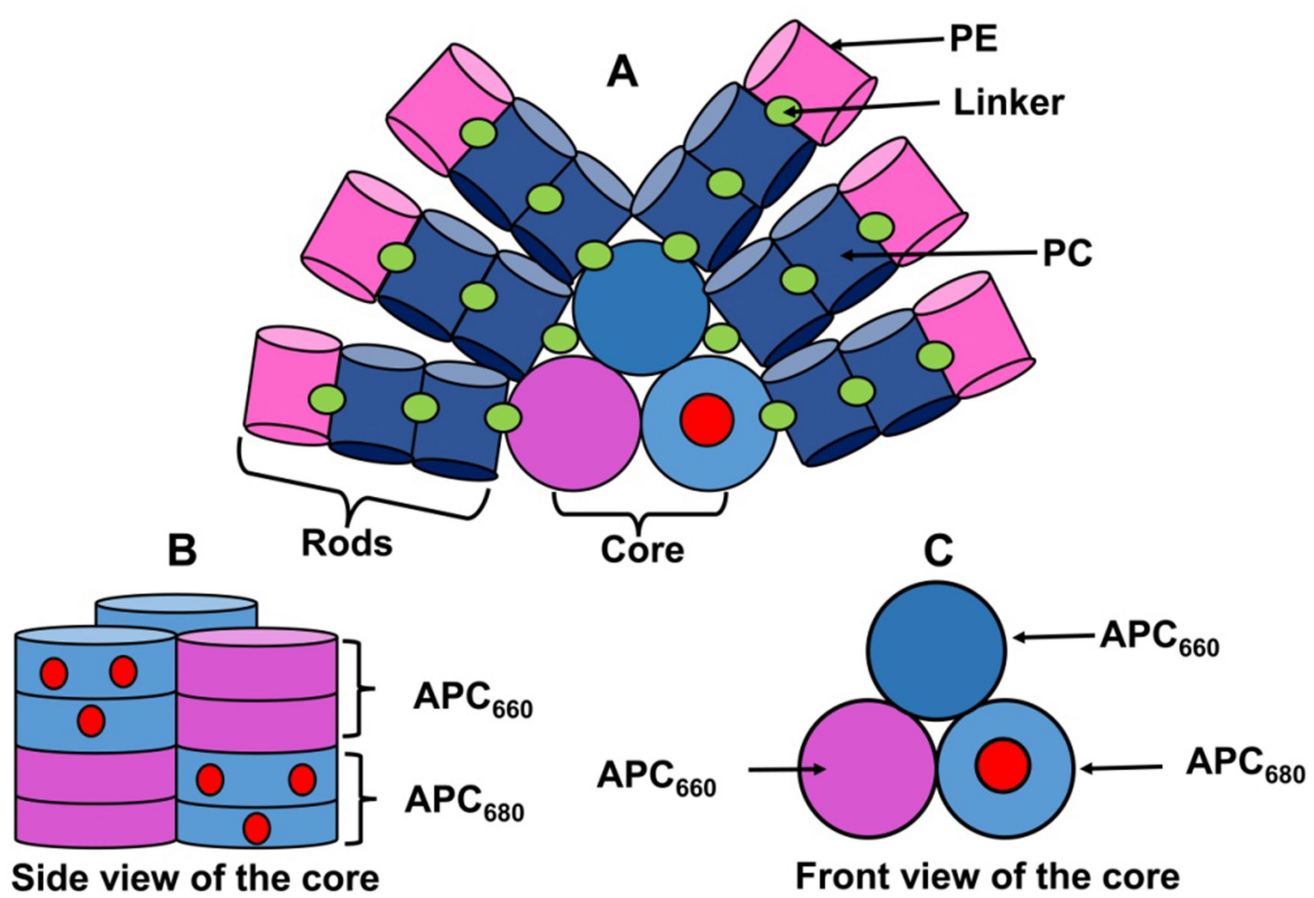
Structure of phycobilisome (PBS) and its components. **(A)** A complete PBS consists of a core and rods. Rods are cylindrical structures composed of PC and/or PE, linked by polypeptides that maintain PBS integrity. **(B)** A side view of the core reveals cylinders, each containing four discs. These discs are composed of two types of APC: APC_660_, found in upper and basal cylinders, and APC_680_, present only in basal cylinders. The antiparallel arrangement of basal cylinders places APC_680_ adjacent to APC_660_. Red dots within APC_680_ indicate variants: one dot represents αAPC_680_ (replaced by αAP-B), and two dots represent αAPC_680_βAPC_680_ (replaced by αL_CM_ and β18.5). **(C)** A front view of the core clearly shows the arrangement of basal and upper cylinders (Stirbet et al., 2019). PE, phycoerythrin; PC, phycocyanin; APC, allophycocyanin.

Unlike most algae and plants, cyanobacteria primarily utilize PBSs as their main light-harvesting complex (LHC) for PSII. For clarity, PBSs can be divided into a “core,” directly attached to the thylakoid membrane at the photosystem, and “rods,” extending distally from the core (Figure 3A). Linker proteins connect the rods to form a 3D hemispherical PBS (Domínguez-Martín et al., 2022). PBSs are substantial structures, ranging from 300 to 800 Å in diameter, with their size inversely related to light intensity (Tamary et al., 2012; Dou et al., 2025). Under very high light, PBS rods can even dissociate, leaving only the core (Mimuro et al., 2008). Nutrient availability also influences PBS structure and composition (Nagarajan et al., 2019). Cyanobacteria exhibit four main PBS morphologies: hemiellipsoidal, block-shaped, bundle-shaped, and hemidiscoidal (Sui, 2021). Hemidiscoidal PBSs are further classified by the number of core cylinders, i.e., bicylindrical or tricylindrical with six rods or pentacylindrical with eight rods (Singh et al., 2015; Sui, 2021). Notably, rod length, composition, and shape are dynamically adjusted in response to environmental cues, including light (Chenu et al., 2017; Gutu and Kehoe, 2012). This adaptability of PBS rods provides cyanobacteria with their characteristic coloration and the ability to efficiently harvest specific light intensities and wavelengths, granting them a competitive advantage over other photosynthetic organisms in diverse light environments.

PBPs, the chromoprotein building blocks of PBS cores and rods, are pigmented heterodimers of α (~17 kDa) and β (~18 kDa) subunits. These subunits covalently bind bilin chromophores, similar to phytochrome in higher plants (Mancini et al., 2018). Unlike chlorophylls, the linear tetrapyrrole chromophores make PBPs water-soluble. The spectral diversity of PBSs arises from the association of these chromophores with the PBP protein moiety (Stirbet et al., 2019). Four main PBP types exist, characterized by their color and light absorption: allophycocyanin (APC; ~650 nm, dark blue), phycocyanin (PC; 620–638 nm, light blue), phycoerythrin (PE; ~565 nm, pink), and phycoerythrocyanin (PEC; ~568 nm, purple). APC and PC bind phycocyanobilin (PCB), with APC having one PCB per α and β subunit, while PC has one per α and two per β subunit (Szalontai et al., 1989; MacColl, 2004). PE binds phycoerythrobilin (PEB) or phycourobilin (PUB) chromophore, absorbing green or blue light, respectively (Dagnino-Leone et al., 2022; Stirbet et al., 2019). PEC binds both PCB and phycoviobilin (PVB) (Zehetmayer et al., 2004). A single rod cylinder consists of a chromophoric (αβ)_6_ hexamer. These acidic cylinders stack via basic linker polypeptides to form rods that connect to the PBS core (Anderson and Toole, 1998).

The core of PBS is made up of trimeric units of α and β subunits of APC, i.e., (αAPCβAPC)_3_ (Sui, 2021). The core is arranged in three piles of cylinders: two at the base and one on top (Figure 3C). The top cylinder connects to the rods via linker proteins. The basal cylinders contain two types of APC, i.e., APC_660_ and APC_680_, having emission peaks at 660 nm and 680 nm, respectively (Jallet et al., 2012). The upper cylinder of the core is made up of four discs of APC_660_ trimers, which transfer energy from the rods to the basal core. Each basal cylinder has four (αAPCβAPC)_3_ discs—two of APC_660_ (αAPC_660_βAPC_660_)_3_ and two of APC_680_ (αAPC_680_βAPC_680_)_3_—which together maintain the structure and function of the PBS (Figure 3B) (Jallet et al., 2012). Out of two discs of APC_680_, in one APC_680_ trimer (αAPC_680_βAPC_680_)_3_, the one αAPC_680_ subunit is replaced by an αAP-B, encoded by the *apcD* gene while another disc of APC_680_ trimer (αAPC_680_βAPC_680_)_3_ has β18.5 subunit (encoded by the *apcF* gene) instead of βAPC, and αL_CM_ subunit (encoded by the *apcE* gene) instead of αAPC in one monomer (αAPC_680_βAPC_680_) of APC_680_ trimer (Jallet et al., 2012) These variations contribute to the structural complexity and functional specialization of the APC trimers (Figure 3B). ApcE (αL_CM_) is essential for stabilizing PBSs by linking core to the TM while its α domain interacts with the PBPs (Sui, 2021). ApcE may regulate state transitions based on the plastoquinone pool’s redox state. ApcD, ApcE, and ApcF mediate energy transfer from APC_680_ to PSII or PSI, with ApcE favoring PSII and ApcD/ApcF primarily exciting PSI during state transitions (Tomar et al., 2024).

### Photosynthetic units (photosystems) in cyanobacteria

**Figure 4:**
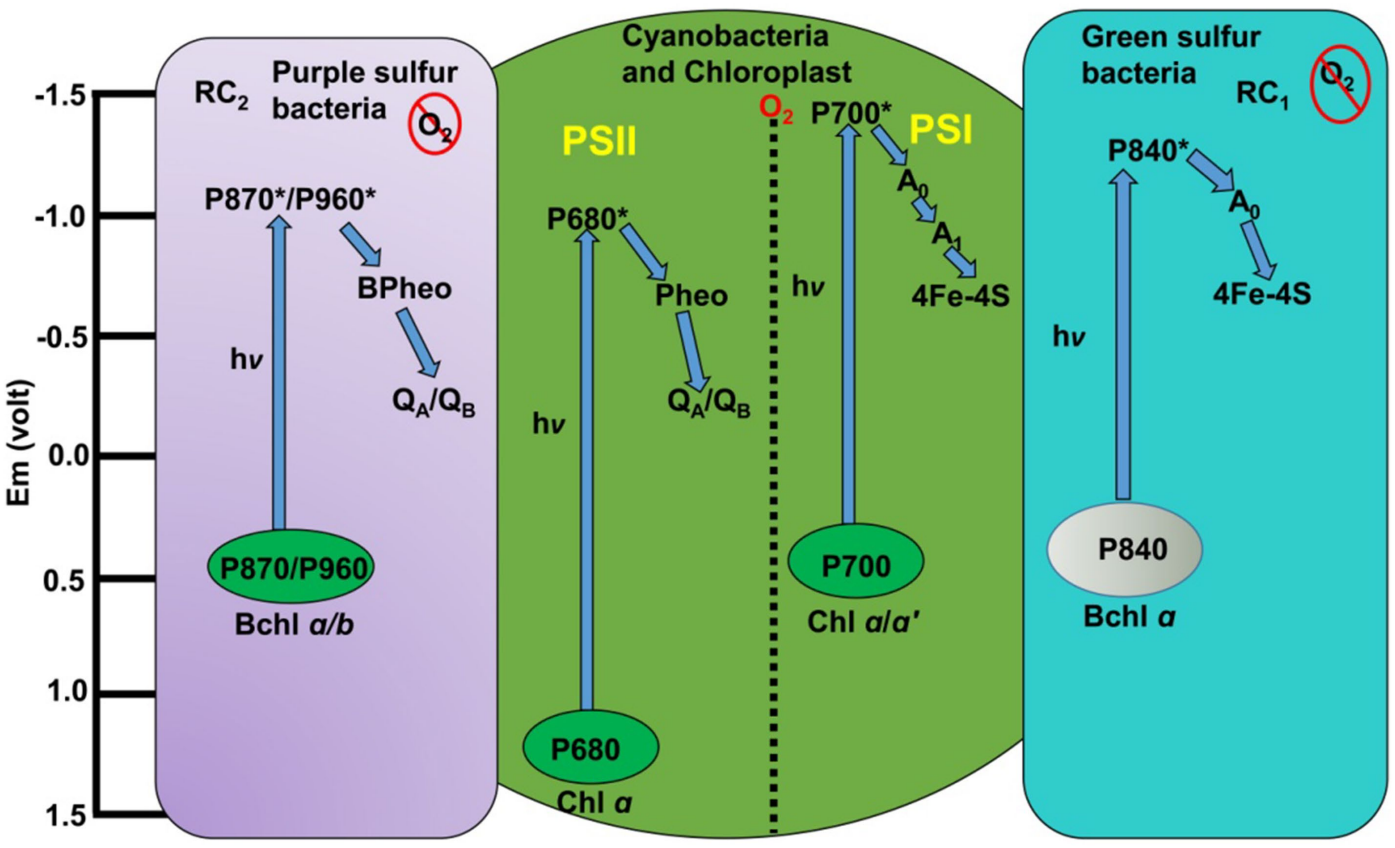
Evolution of reaction centers in photosynthetic bacteria. The purple rectangle represents the quinone-type reaction center (RC) of purple sulfur bacteria, where quinone is the terminal electron acceptor. The blue rectangle depicts the FeS-type RC of green sulfur bacteria, with an FeS center as the final electron acceptor. An evolutionary event brought these two RC types together in a single organism, forming a complete electron transport chain (ETC) that produces both ATP and NADPH in cyanobacteria (green circle), the first oxygenic photoautotrophs (Shevela et al., 2013). Em, electrode potential in volt; RC_2_, reaction center 2; RC_1_, reaction center 1; h*ν*, light; Bchl, bacteriochlorophyll; Chl, chlorophyll; P870/P960, P680, P700, P840, pigment 870/960, 680, 700 and 840 indicates reaction centers; P870*/960*, 680,700 and 840* indicates excited state of all reaction centers; Bpheo, bacteriopheophytin; Pheo, pheophytin; Q_A_/Q_B_, quinone A/quinone B; PSII and PSI, photosystem II and photosystem I; A_0_, A_1_, electron acceptors of PSI; 4Fe-4S, iron-sulfur clusters; O_2_, oxygen; PS, photosystem; hν, light of specific wavelength.

Photoautotrophs possess natural photocells, termed photosystems (PSs), to perform light absorption and photochemical reactions. These reactions convert light energy into chemical energy, fueling the organism’s survival, maintenance, and reproduction. This energy also forms the base of the food web. A photosystem comprises a light-capturing antenna and a reaction center, orchestrating energy excitation, transfer, and photochemistry. Anoxygenic green and purple sulfur bacteria were the first to evolve strategies for harvesting and converting light energy into chemical bond energy (Blankenship, 2010; Hu et al., 1997; Larkum et al., 2018). These prokaryotes independently developed two distinct types of reaction centers—the site of photochemistry—distinguished by their terminal electron acceptors (Figure 4). These are the FeS-type (type I) reaction center found in green sulfur bacteria and the quinone-type (Q-type or type II) reaction center of purple sulfur bacteria (Blankenship, 2010). Green and purple sulfur bacteria utilize H_2_, H_2_S, or S as electron sources for photochemistry (Martin et al., 2018). Given Earth’s early reducing atmosphere, these sulfur bacteria were likely the planet’s first photoautotrophs. Their reaction centers served as the foundation for the evolution of modern oxygenic photosynthesis (Ohashi et al., 2010). The limited availability of H_2_, H_2_S, and S, coupled with the abundance of water, likely drove the selection pressure for the evolution of the oxygen-evolving complex (Xiong and Bauer, 2002; Tomitani et al., 2006). Consequently, it is plausible that purple and green sulfur bacteria evolved PSII and PSI, respectively, that eventually leads to evolution of cyanobacteria capable of oxygenic photosynthesis (Sharon et al., 2009; Govindjee and Shevela, 2011).

#### Photosystem II (PSII): structure and function

Crystal structures of PSII solved at various resolutions have provided definitive insights into its physicochemical properties (Shen, 2015; Zouni et al., 2001). Notably, a 1.9 Å resolution structure revealed the dynamic interactions of amino acid side chains with metallic and organic cofactors (pigments) during the light reactions (Umena et al., 2011). The following section will detail the structure of PSII. PSII, a ~ 700 kDa dimeric multisubunit membrane protein supercomplex also known as water/plastoquinone oxidoreductase (Wegener et al., 2015; Shen, 2015), presents challenges for intact isolation, leading to studies on its monomeric (~350 kDa) form. The arrangement of PSII subunits facilitates unidirectional excitation energy and electron transfer from lower to higher redox potentials (Stirbet et al., 2019). Spectrally, PSII comprises two pigment-protein complex regions: the peripheral antenna, which harvests light energy and transfer it to the reaction center or core antenna (Zuber, 1986; Stirbet, 2013).

**Figure 5:**
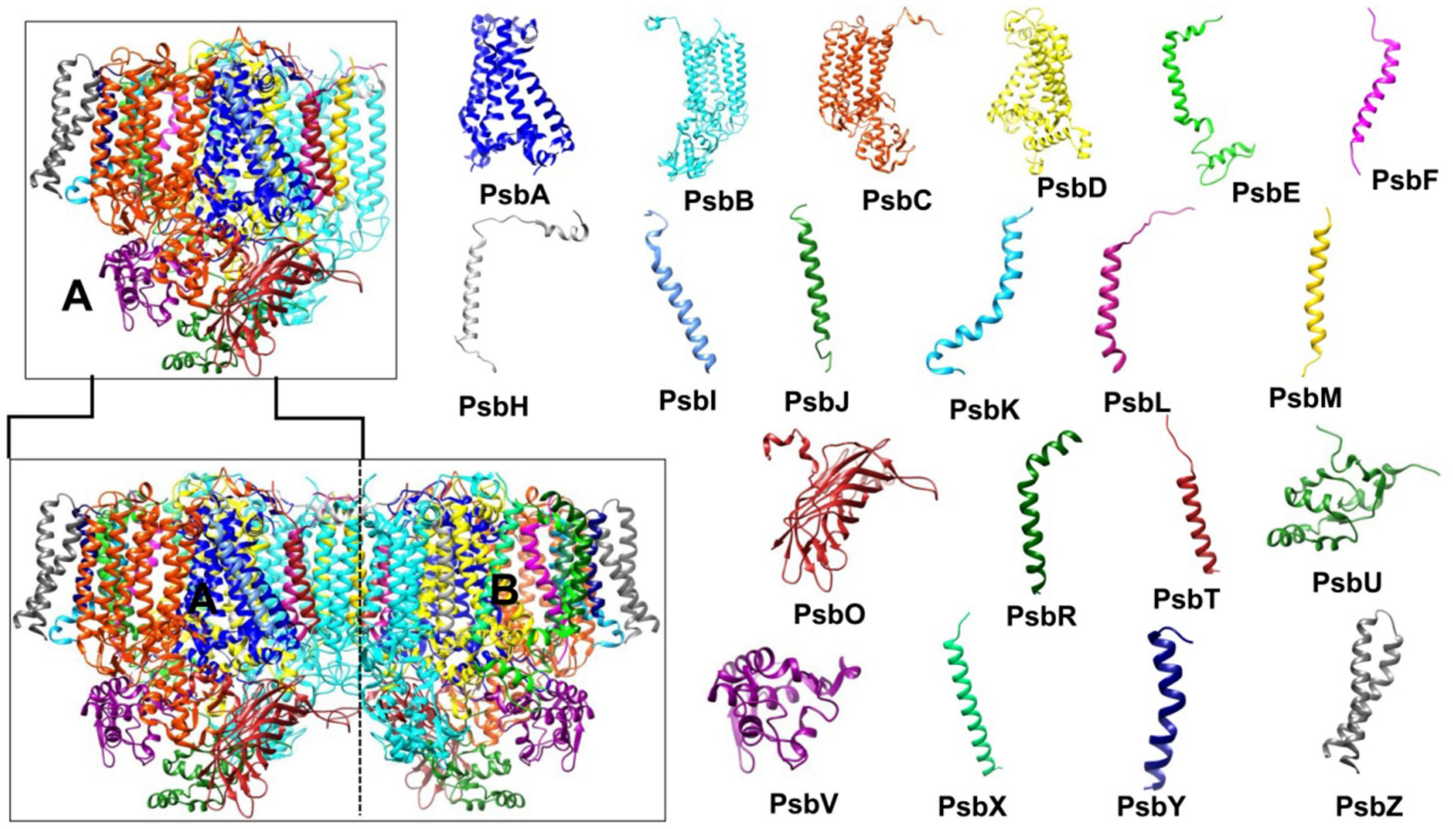
Photosystem II (PSII) structure (PDB ID: 7YQ7, 1.9 Å resolution). The bottom left shows the PSII dimer (subunits A and B), while the top left displays the PSII monomer (subunit A). The right side illustrates the structure of the 20 subunits comprising the PSII monomer. PSII, photosystem II.

Cyanobacteria were crucial in elucidating the conserved molecular organization of core antennae, essential for photochemistry in all oxygenic photoautotrophs. Molecular analysis of PSII core antennae reveal that its axial symmetry involve two distinct polypeptides, CP43 and CP47, which bridge the reaction center and antennae for efficient excitation energy transfer (Umena et al., 2011). The core itself consists of a D1 (PsbA) and D2 (PsbD) heterodimer, binding redox-active cofactors near the reaction center for electron transfer. The antennae are composed of Chl-binding proteins CP43 (PsbC) and CP47 (PsbB), binding 13–16 Chls and 4–5 β-carotenes (Stirbet et al., 2019). The 1.9 Å resolution crystal structure of the PSII monomer (Figure 5) showed it comprises 17 integral membrane protein subunits (large and low molecular mass) and three extrinsic protein subunits on the luminal side (Umena et al., 2011). The large molecular mass subunits are integral membrane proteins, while the low molecular mass subunits include the (PsbE) and (PsbF) subunits of cytoplasmic Cytochrome *b_559_* (Cyt *b_559_*) (Loll et al., 2007). Although Cyt *b_559_* does not participate in PSII photochemistry, it maintains the structural integrity of the PSII core (Chiu et al., 2022). Three extrinsic membrane proteins, PsbO, PsbV, and PsbU, are attached to the luminal surface. These proteins protect the oxygen-evolving complex from redox-active cofactors of the electron transport chain and maintain the integrity of the large molecular mass subunits (D1, D2, CP43, and CP47) because loops of these integral proteins extend into the thylakoid lumen (Iwai et al., 2010; Shen, 2015). Thus, extrinsic membrane proteins act as a protective cap for these loops.

The D1 and D2 proteins are symmetrically positioned within the transmembrane region, forming the D1/D2 heterodimer, with two branches binding redox-active cofactors (Umena et al., 2011). These cofactors include six Chl *ɑ* molecules (P_D1_, P_D2_, Chl_D1_, Chl_D2_, Chl_ZD1_, Chl_ZD2_, named for their D1 or D2 branch association), two pheophytins (Pheo_D1_ and Pheo_D2_), a tightly bound Q_A_ (on D2), and a loosely bound Q_B_ (on D1). PQ molecules, Q_A_ and Q_B_, are bound to specific amino acids of D2 and D1, respectively. A non-heme iron, equidistant between Q_A_ and Q_B_, facilitates sequential electron transfer in the photosynthetic electron transport chain (Umena et al., 2011). Additionally, two β-carotenes, four manganese ions, three or four Ca^2+^ ions (one bound to Mn_4_CaO_5_), three chloride (Cl^−^) ions, and one carbonate (CO_3_^2−^) or bicarbonate (HCO_3_^−^) ion are bound near the non-heme iron, participating in Q_B_ protonation during its reduction by Q_A_ (Shevela et al., 2012).

#### Photosystem I (PSI): structure and function

**Figure 6:**
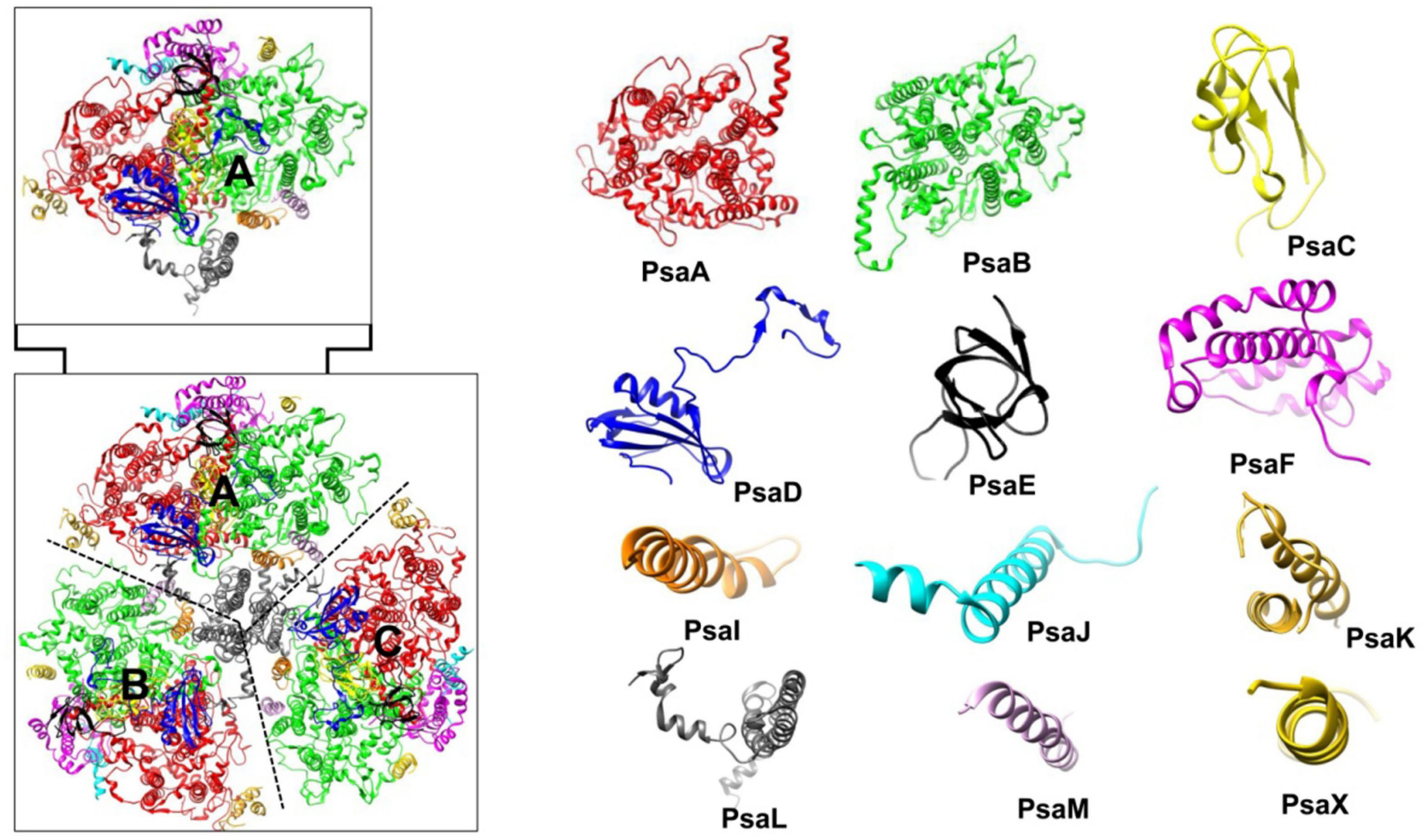
Photosystem I (PSI) Structure (PDB ID: 1JB0, 2.5 Å resolution). The bottom left shows the PSI trimer (subunits A, B, and C), while the top left displays the PSI monomer (subunit A). The right side illustrates the structure of the 12 subunits comprising the PSI monomer. PSI, photosystem I.

Jordan et al. (2001) determined the 2.5 Å crystal structure of PSI, revealing its threefold symmetry as a trimer (Figure 6). The PSI monomer comprises 12 subunits (PsaA–F, PsaI–M, and PsaX) and 127 cofactors: 96 Chls, 22 Cars, 2 phylloquinones, PhQ (A_1A_, A_1B_), 3 [4Fe-4S] clusters, 1 putative Ca^2+^, and 4 lipid molecules (Jordan et al., 2001; Nagao et al., 2023; Stirbet et al., 2019). PSI, also known as plastocyanin (Pc)/ferredoxin (Fd)-oxidoreductase, receives electrons from the mobile carrier Pc (transferring electrons from the Cyt *b_6_f* complex) and donates them to Fd, the terminal electron acceptor of the Z-scheme, which then reduces Fd-dependent NADPH reductase (FNR) (Stirbet et al., 2019).

Of the 12 PSI subunits, PsaL, PsaX, and PsaM are absent in higher plants, with PsaM and PsaL specifically driving PSI trimer or tetramer formation in cyanobacteria (Nagao et al., 2023; Chen et al., 2022). In certain thermophilic and heterocyst-containing cyanobacteria, additional copies of PsaL enable the formation of PSI tetramers (Li et al., 2014). The PSI tetramer is particularly significant for its thermostability, which makes it an ideal target for engineering the PSI. In higher plants, PsaH prevents PSI trimer formation (Li et al., 2014), suggesting that PSI might form trimers under specific environmental conditions, even without PsaL and PsaM, though these two proteins enhance trimer stability.

The PSI core antenna is primarily composed of PsaA and PsaB subunits. Similar to CP47 and CP43 in PSII, the Chl *ɑ* molecules linked to the six-helix domains in the N-terminus of PsaA and PsaB facilitate light absorption (Barber et al., 2000). Additionally, 10 Chls are associated with subunits PsaG, PsaK–M, PsaX, and a phosphatidylglycerol molecule. Due to trimeric or tetrameric nature of PSI, it has a high number of associated Chls and Cars (60–90 Chls in the inner antenna are associated with Cars, with most β-carotene linked to long-wavelength Chls) (Karapetyan et al., 1999; Karapetyan et al., 2014). In addition to their light absorbing and photoprotective properties, Cars aid in the assembly and stabilization of the pigment-protein complex (Wang et al., 2004).

## Photochemistry and photosynthesis-respiration interplay

Photochemistry is a rapid natural chemical reaction fundamental to all photoautotrophs. Photosynthesis results from two sequential chemical reactions that take place in the TM and stroma of chloroplast. However, in cyanobacteria, it takes place in the TM and carboxysome-cum-cytoplasm (Rohnke et al., 2018). The first step involves photochemistry while second step involves enzymatic reduction of CO_2_ using the byproducts of photochemistry, i.e., NADPH and ATP. Spectral analysis reveals that Chl molecules absorb maximally in the blue and red regions. However, Chl *ɑ* molecules with red and far-red absorption maxima were evolutionarily favored to form the reaction centers of both PSs due to their capacity to store significant energy for charge separation (Shevela et al., 2013). Thus, Chl molecules in the reaction center are replaced to optimize absorption based on available light wavelengths. Antennae also contain blue-absorbing Chl *ɑ* molecules. These lower-wavelength absorbing Chls are positioned away from the reaction center to maximize light capture while minimizing excitation pressure (Björn et al., 2009).

Upon absorbing blue light (450–500 nm), Chl molecules reach a highly unstable highest excitation state. They rapidly lose some energy as heat to transition to a lower, more stable excited state, from which they can release energy via fluorescence, heat dissipation, resonance energy transfer, or photochemistry (Björn et al., 2009). In plants, light-harvesting complexes, and in cyanobacteria, the PSII inner core antenna, arrange Chls around the reaction center to facilitate energy transfer through excitation or resonance (Shevela et al., 2013; Romero et al., 2010). The TM houses two PSs where non-cyclic electron flow (Z-scheme) involving both PSII and PSI produces NADPH and ATP (non-cyclic photophosphorylation). Cyclic electron transport, involving only PSI, generates ATP (cyclic photophosphorylation) (Bendall and Manasse, 1995; Petrouleas and Crofts, 2005; Kok et al., 1970; Arnon, 1984; Lea-Smith et al., 2016; Giera et al., 2009; Hill and Rich, 1983; Albertsson, 1995; Tikhonov, 2014). Unlike plants, cyanobacteria lack peripheral antennae but possess PBSs to capture light poorly absorbed by PSs. Chl fluorescence (ChlF) spectra, showing strong emission at 685–695 nm (PSII) and 715–730 nm (PSI), demonstrate PBSs’ role in delivering excitation energy to PSII and PSI via state transition (Rijgersberg and Amesz, 1980). The parallel increase in PSI and PSII fluorescence yield with a decrease in PBS fluorescence over time, evident in time-resolved 77 K fluorescence spectra, confirms PBS-PS interaction (Ueno et al., 2017).

Despite their arrangement, PSs can likely efficiently absorb excitation energy from PBSs (David et al., 2014). Three models explain this transfer: (1) direct PBS-PSI energy transfer (Akhtar et al., 2022), (2) simultaneous PBS interaction with both PSII and PSI forming a PBS-PSII-PSI supercomplex (Liu et al., 2013), and (3) spillover, where a PBS-PSII supercomplex transfers energy to PSI (Ueno et al., 2017; Calzadilla and Kirilovsky, 2020). Cyanobacterial TMs uniquely house both photosynthetic and respiratory electron transport chains (Figure 2), converging at the PQ pool, Pc, and the cytochrome *b_6_f* complex. The TM contains succinate dehydrogenase (SDH), NAD(P)H dehydrogenases (NDH), and terminal oxidases like Cyd and Cox. Active in darkness or low light, SDH oxidizes succinate, and NDH oxidizes NADPH (Mullineaux, 2014). The presence of respiratory complexes on the TM means PSI competes with respiratory carriers for electron flow in photochemistry. The PQ pool can be reduced by linear photosynthetic electron transport or by respiratory electron transport from NADPH (via NDH-1) and succinate (via SDH) (Mullineaux, 2014; Willeford et al., 1989). Under high light, Cox competes with P_700_ for electrons from Pc or Cyt c_6_ (Ermakova et al., 2016). Changes in PSI stoichiometry under high light can divert electrons from PSII to Cox. Conversely, blocking terminal oxidases, as in darkness, allows respiratory electrons to reduce the acceptor side of PSII (Nikkanen et al., 2021; McDonald et al., 2011). Thus, respiratory electron carriers serve as an electron source in low light and a sink in high light, preventing photoinhibition (Nikkanen et al., 2021; McDonald et al., 2011).

## Light regime and cyanobacterial photosynthetic machinery

Photosynthetic organisms, including cyanobacteria, inhabit environments characterized by fluctuating light conditions, encompassing both low and high intensities of PAR and UVR. These variations, inherent in day-night cycles, and seasonal and spatial changes, significantly impact the overall fitness of these organisms by affecting fundamental cellular processes and causing damage to crucial biological molecules (Mondal et al., 2024; Singh et al., 2010). The following sections will summarize the detrimental effects of different light regimes and the photoacclimation strategies cyanobacteria have evolved to thrive in such dynamic environments.

### High and low PAR: photosynthetic consequences

Light intensity in natural ecosystems fluctuates seasonally and diurnally, exerting differential effects on photoautotroph fitness. High PAR can damage the D1 protein of PSII, a phenomenon termed photoinhibition (Long et al., 1994). Photoinhibition triggers non-photochemical quenching (NPQ), driven by Cars associated with LHCII in response to low TM pH in higher plants and algae (Li et al., 2000; Calzadilla and Kirilovsky, 2020). This pH decrease also induces state transitions (state 2) of LHCII and the production of ROS (Bennett, 1991; Allen, 2003). To counteract increased ROS, higher plants and algae elevate the levels and activity of antioxidative enzymes, antioxidants, and Cars for ROS mitigation (Middleton and Teramura, 1993).

Despite their long evolutionary history in dynamic light environments, cyanobacteria are negatively impacted by abrupt changes in light intensity. High-intensity blue-green or white light triggers NPQ in these organisms (Gorbunov et al., 2011). Elevated PAR levels induce oxidative stress, or photooxidation, where the over-accumulation of Fd, a strong reductant, readily reduces oxygen molecules, producing superoxide and peroxide radicals that can damage biological molecules (Latifi et al., 2009). Furthermore, the triplet state of Chl *ɑ* can transfer excitation energy to molecular oxygen, forming singlet oxygen (^1^O_2_^∗^), which can then transfer its energy to lipid molecules. This singlet oxygen can disrupt the integrity of the TM and potentially lead to the disassembly of the oxygen-evolving complex (Asada, 1999). Additionally, increased singlet oxygen levels activate the production of high-light inducible proteins (HLiPs) (Komenda and Sobotka, 2016). Conversely, cyanobacterial cells acclimated to low light conditions are particularly vulnerable to rapid D1 protein damage upon sudden increase in light intensity. Consequently, low-light-grown cultures require extended periods to acclimatize to even slight increases in irradiance (Zhou et al., 2024). Furthermore, low light intensity also promotes state transitions, a phenomenon discussed in detail in a subsequent section.

### UVR and photosynthetic impairment

UVR impairs photosynthesis through multiple mechanisms beyond photoinhibition. It negatively impacts the activity of ribulose-1,5-bisphosphate carboxylase/oxygenase (RuBisCO), CO_2_ intake, and the oxygen-evolving complex (Watanabe et al., 2023). Conversely, low-intensity UV-A (315–400 nm) radiation can enhance photosynthesis and biomass accumulation in certain cyanobacteria (Grettenberger et al., 2024). Furthermore, UVR exposure induces the generation of ROS at various sites within the photosynthetic and respiratory electron transport chains, as well as during diverse metabolic reactions (Napaumpaiporn et al., 2024; Noyma et al., 2015).

UVR exerts numerous damaging effects on cyanobacterial cells, including photobleaching of photosynthetic pigments and a significant decrease in protein content. The reduction in absorption and fluorescence emission spectra of PBPs upon UVR exposure is linked to decreased PBP content and the disassembly of PBSs (Six et al., 2007). UVR also inhibits CO_2_ fixation and damages DNA by inducing the formation of thymine dimers and other photoproducts (Singh et al., 2023a). Prolonged UVR exposure can impair nitrogenase activity, severely affect heterocyst and akinete differentiation, and reduce the gliding motility of filamentous cyanobacteria (Rastogi et al., 2014). Moreover, UVR inhibits the photophobic and phototactic responses of cyanobacteria, thereby compromising their ability to avoid damaging radiation and ultimately leading to photodamage and cell death (Singh et al., 2023a).

## Acclimation strategies to spectral quality

Found in diverse ecological niches—oceans, seas, freshwater, glaciers, and land—cyanobacteria are exposed to temporally, diurnally, and seasonally changing light regimes. Consequently, they have evolved various strategies to adjust their position in the water column and optimize their photosynthetic machinery, including light-harvesting, for efficient photon utilization. Cyanobacteria possess broad range of photosensors that sense different wavelengths/quality/color of light, enabling them to initiate various photoacclimatization and/or developmental processes (Gupta et al., 2023). These photosensors can sense different part of the solar spectrum, including visible light, UVR and far-red light (Bandara et al., 2021; Gupta et al., 2023). Cyanobacterial photosensors belong to the cyanobacteriochrome (CBCR) phytochrome superfamily. Phytochromes (Phys) possess a tripartite N-terminal photosensory domain, comprising PAS, GAF, and PHY domains (Bandara et al., 2021; Gupta et al., 2023), also known as the photosensory core module. The GAF domain (cGMP phosphodiesterase, adenyl cyclase, and FhlA) binds linear tetrapyrrole (bilin) pigments via a thioether bond to detect light signals (Xu et al., 2020). Phytochromobilin bound to the GAF domain can exist in different configurations (5-Z,syn, 10-Z,syn, and 15-Z,anti) in its dark/low-light protonated form (Pr). Conversely, red-light-rich, high-light environments induce rotation around the C15–C16 double bond, resulting in the 15-E,anti configuration (Pfr form). The activated Pfr form of Phy triggers the expression of various genes by entering the nucleus (Bandara et al., 2021).

In contrast to Phys, CBCRs exhibit greater diversity in sensing a wider spectrum of light, from near-UV or visible to far-red, utilizing PCB (Bandara et al., 2021). Like Phys in higher plants, CBCRs possess one or more GAF domains that efficiently bind bilins via thioether linkages (Bandara et al., 2021; Gupta et al., 2023). The molecular evolution of the GAF domain under varying light conditions has resulted in the spectral diversity of CBCRs, enabling cyanobacteria to perceive different light qualities (Bandara et al., 2021; Gupta et al., 2023). The nature of the thioether bond between phycobilin and the GAF domain dictates the spectral properties of CBCRs; for example, a two-thioether linkage allows absorption of near-UV, violet, and blue light (Bandara et al., 2021). The spectral diversity of cyanobacteriochromes (CBCRs) makes them valuable tools in synthetic biology and optogenetics. Their ability to enable light-mediated modulation of gene transcription allows for easy tracking of genetic activity within cells and tissues by combining different CBCRs (Ariyanti et al., 2021; Gupta et al., 2023). Additionally, various CBCRs can be used to promote phototaxis in engineered prokaryotic and eukaryotic strains in response to specific light signals (Ariyanti et al., 2021).

Based on the perceived light color, the corresponding signal is transduced by the two-component signaling system of CBCRs to trigger cellular responses (Gupta et al., 2023). One ecological significant phenomenon in cyanobacteria is CA, which is a wavelength-dependent adjustment in the protein and/or pigment composition of PBSs. CA allows cyanobacteria to optimize their fitness and photosynthetic efficiency in dynamic light environments (Montgomery, 2017; Kumar et al., 2019; Mondal et al., 2024). The following sections will briefly outline different light conditions under which cyanobacteria employ CA to maximize their photosynthetic fitness by modifying their light-harvesting apparatus.

A common type of CA observed in some marine cyanobacteria involves adjustment to blue and green light, which penetrate deeper into clean ocean and sea environments. Under blue light condition, the chromophore phycourobilin (PUB) associates with phycoerythrin (PE), enabling the organism to absorb blue light and appear orange (Carrigee et al., 2020). Conversely, in green light environment, the chromophore phycoerythrobilin (PEB) is incorporated into the PE apoprotein, maximizing green light absorption and giving the organism a brick-red color (Gupta et al., 2023; Mondal et al., 2024). This dynamic chromophore attachment to PE allows these cyanobacteria to efficiently harvest the prevalent wavelengths: PUB under blue light and PEB under green light. While green light is typically not utilized by photosynthetic organisms, PEB allows certain cyanobacteria to perform photosynthesis using green light. Similarly, although cyanobacteria generally rely on PBSs for PSII excitation, limiting blue light utilization (Luimstra et al., 2018), the PUB chromophore provides a unique advantage to some marine species to efficiently use blue light for photochemistry.

CA in response to red and green light is well-documented in the cyanobacterium *Fremyella diplosiphon* where change in the levels of PC and PE in PBS take place under red and green light growth conditions, respectively (Montgomery, 2017; Mondal et al., 2024). CA enables cyanobacteria to acclimatize to far-red light environment (Wiltbank and Kehoe, 2019). The far-red shifting of PBS and occurrence of Chl *d* and Chl *f* in the PS is the characteristics features of CA in *Halomicronema hongdechloris*, *Chlorogloeopsis frtischii*, *Leptolyngbya* sp. strain JSC-1. Red/far-red CBCRs control the accumulation of proteins that constitute red-shifted PSII and far-red shifted PBSs (Mondal et al., 2024; Montgomery, 2017; Wiltbank and Kehoe, 2019).

## Acclimation strategies to light intensity

The detrimental effects of light on photosynthetic organisms are particularly relevant in the context of global climate change. Fluctuating light conditions, coupled with other abiotic stressors like salinity, temperature, nutrient availability, and UVR, can have severe consequences. Consequently, understanding the photoprotective mechanisms evolved by these ancient organisms is crucial for addressing challenges associated with global climate change. The following sections will explore various photoprotective strategies employed by cyanobacteria to mitigate the damaging effects of PAR.

### Non-photochemical quenching (NPQ)-based photoprotection

NPQ encompasses processes that dissipate excess excitation energy as heat, rather than through fluorescence or photochemistry. This ensures efficient functioning of PSII under high irradiance. The following are various mechanisms by which cyanobacteria release surplus excitation energy in the form of heat to safeguard their photosynthetic machinery.

#### Orange carotenoid protein (OCP)

High light intensity induces NPQ in the peripheral antennae of green algae and plants, where a significant portion of energy is released as heat (Asada, 1999). Cars quench excess energy without forming triplet states, thus also acting as ROS scavengers. Cyanobacteria utilize an orange carotenoid protein (OCP^O^)-based NPQ mechanism. Upon absorbing blue-green light, the OCP^O^ form converts to a red carotenoid protein (OCP^R^) form. OCP^R^ dissipates excessive high-light energy absorbed by PBSs as heat, ensuring that only an optimal amount of energy is transferred to the reaction centers (Wilson et al., 2022; Domínguez-Martín et al., 2022; Kirilovsky, 2007).

Nearly all cyanobacterial strains possess OCP, and OCP-deficient mutants exhibit increased susceptibility to photodamage. The gene encoding OCP is constitutively expressed and further upregulated under high light conditions (Kirilovsky and Kerfeld, 2012). In *Synechocystis*, OCP is encoded by the *slr1963* gene. However, it is absent in the freshwater *Synechococcus elongatus* PCC 7942 and the thermophile *Thermosynechococcus elongatus* (Kirilovsky and Kerfeld, 2012; Tiwari et al., 2024). Consequently, these OCP-lacking cyanobacteria are sensitive to high-light radiation. Nevertheless, they can protect themselves by reducing their PBPs content to mitigate damage from excess light. These organisms can also functionally disconnect their PBSs, a phenomenon more common under iron-limiting conditions (Kirilovsky and Kerfeld, 2012; Tiwari et al., 2024).

**Figure 7:**
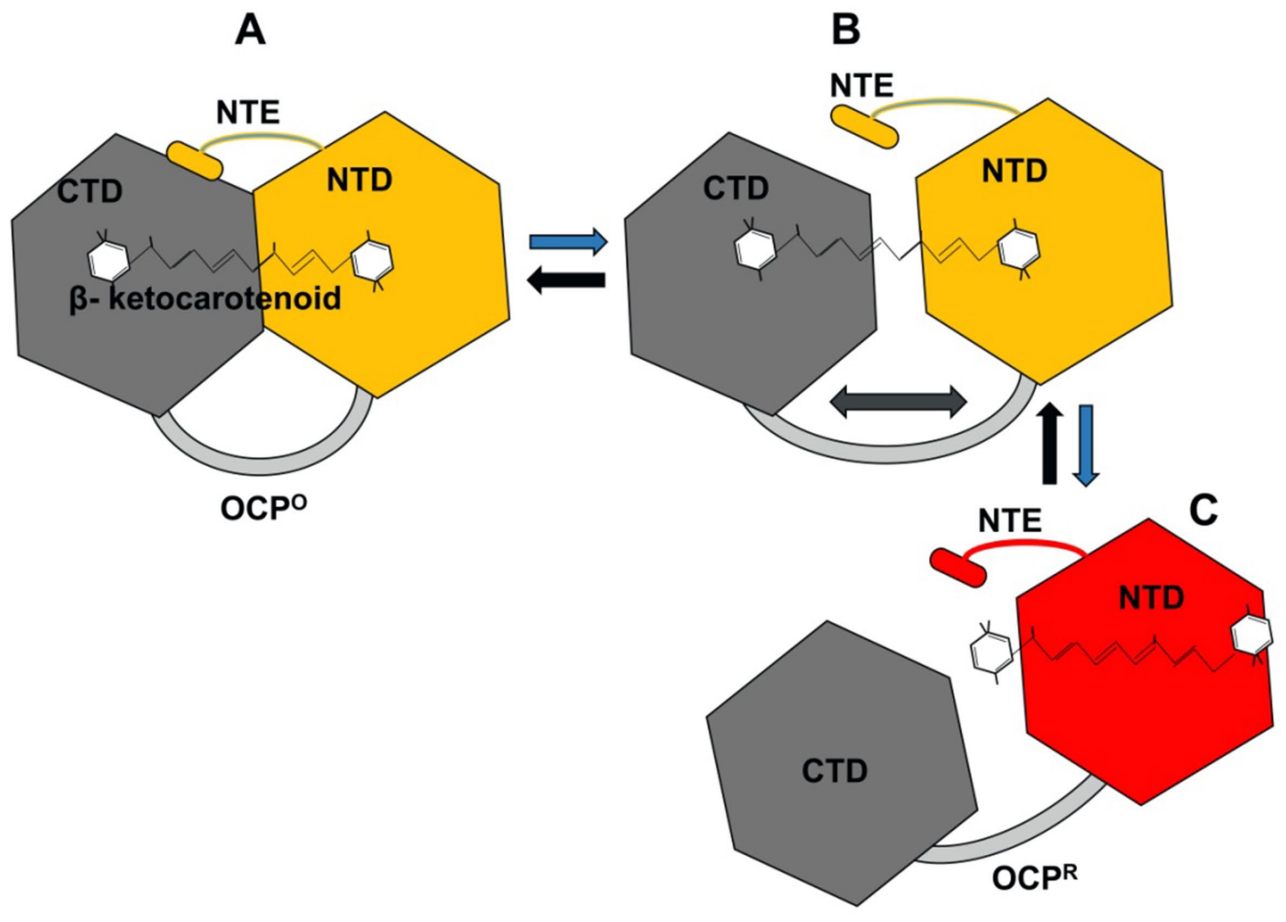
Conformational states of OCP under light and dark conditions. Blue arrows indicate blue/high light conditions, while black arrows show reversion to the OCP^O^ state in darkness. **(A)** In the inactive OCP^O^ state, the NTE is attached to the CTD, and the β-ketocarotenoid spans both domains. **(B)** Absorption of blue/high light induces a conformational change in the β-ketocarotenoid, leading to OCP activation where the NTE detaches from the CTD. The NTD and CTD remain linked by a loop. **(C)** Complete dissociation of the NTD and CTD occurs, with the β-ketocarotenoid fully translocated into the NTD to bind with APC and regulate excitation energy transfer to the reaction center (Stirbet et al., 2019). CTD, C-terminal domain; NTD, N-terminal domain; NTE, N-terminal extension; OCP^O^, orange carotenoid protein (orange form); OCP^R^, orange carotenoid protein (red form).

OCPs are water-soluble proteins of approximately 35 kDa that non-covalently bind a keto-carotenoid molecule, typically 3′-hydroxyechinenone (3-hECN) or its homologs (Domínguez-Martín et al., 2022). Cyanobacteria possess at least three paralog families of OCPs: OCP_1_, OCP_2_, and OCP_X_, with OCP_1_ being the most prevalent (Slonimskiy et al., 2022). Localized on the cytoplasmic side of the thylakoid membrane near PBSs, OCPs consist of two domains (Figure 7): an α-helical N-terminal domain (NTD) and an α/β C-terminal domain (CTD). The CTD, belonging to the nuclear transport factor-2 superfamily, is connected to the NTD by a flexible linker of approximately 25 residues, which is part of an unstructured intrinsically disordered protein region (Leverenz et al., 2014; Leverenz et al., 2015).

In darkness or low light, the orange (inactive) form of OCP predominates. However, high-intensity blue-green light induces a conformational change in the bound carotenoid molecule, leading to the conversion of inactive OCP^O^ into a metastable OCP^R^. This OCP^R^ then interacts with PBSs to perform NPQ by dissipating their excess excitation energy (Domínguez-Martín et al., 2022; Prabha et al., 2025). Recent findings indicate that OCP^R^ forms a dimer to interact with PBSs. However, this interaction is selective, requiring a suitable conformation of the PBSs, meaning not all PBSs can interact with the OCP^R^ dimer (Domínguez-Martín et al., 2022).

In the presence of Cu^2+^, photoactivation of OCP results in its stabilization in the red form (OCP^R^). This occurs because Cu^2+^ binding to a cysteine residue prevent the return of protein to its inactive orange state (Liu et al., 2016). Upon activation, OCP undergoes a structural change where the NTD and CTD separate while remaining connected by a linker loop (Figure 7B). Subsequently, the keto-carotenoid spanning both domains detaches from the CTD and shifts 12 Å into a cavity within the NTD (Figure 7C), forming OCP^R^ (Thurotte et al., 2015; Maksimov et al., 2017a, 2017b). This detachment during photoactivation involves either β-ionylidene ring rotation or a transient keto-enol shift (Maksimov et al., 2015; Bandara et al., 2017). Finally, OCP^R^ interacts with the core hexamer of PBSs, ApcC, becoming buried within the terminal hexamer of the basal core cylinder (Harris et al., 2018; Gwizdala et al., 2018).

Thus, structural changes in PBSs influence excitation energy transfer kinetics. Spectroscopic analysis during OCP docking indicates a temporary decoupling of some PBS rods (Bar Eyal et al., 2017), while another study proposes a PBS conformational change to facilitate OCP^R^ interaction (Domínguez-Martín et al., 2022). Under low light or dark conditions, Fluorescence Recovery Protein (FRP), encoded by *slr1964* in *Synechocystis* sp. PCC 6803, removes OCP^R^ from PBSs (Liu et al., 2018). This action halts the process of NPQ and allows the fluorescence of the PBSs to recover. This ~13 kDa water-soluble protein, lacking a chromophore, primarily exists as a functional dimer, transitioning from an inactive tetramer (Thurotte et al., 2017). FRP specifically interacts with the CTD of OCP^R^ to accelerate its detachment from PBSs and promote its deactivation to OCP^O^ (Thurotte et al., 2017). However, excess Cu^2+^ inhibits FRP’s interaction with OCP^R^ by locking OCP in the OCP^R^ form, potentially aiding the organism in tolerating high-light conditions in Cu^2+^ stressed cells (Liu et al., 2018; Lou et al., 2019). In summary, under high light conditions, the OCP cuts off the excitation energy transfer from PBSs to PSII, thereby lowering the photochemical quantum efficiency of PSII. While this strategy protects PSII from photodamage, it also reduces the overall photosynthetic efficiency of cells exposed to high light.

#### Iron-starvation inducible protein (IsiA)

Fluctuating environments can disrupt the redox balance in cyanobacteria (Jeanjean et al., 2003; Havaux et al., 2005). Under high light and iron depletion, the Chl-binding TM protein IsiA forms photoprotective rings around PSI and its antenna (Zhang et al., 2010). It is homologous to CP43 (PsaC subunit in PSII), and therefore, also called as CP43`. These PSI_3_-IsiA_18_ supercomplexes (6 IsiA per PSI monomer) dissipate excess light energy as heat (Reinot et al., 2022). Combined high light and iron starvation also lead to IsiA-PSI-PSII supercomplex formation, a condition where PBSs become functionally disconnected (Wang et al., 2010). However, under iron stress and strong light, free PBSs can couple with IsiA, which prevents damage from dissociated PBSs (Joshua and Mullineaux, 2005). In *Synechocystis* sp. 6,803 and *Synechococcus elongatus* PCC 7942, IsiA protects PSI and its antenna under iron-deficient and high-light conditions (Nodop et al., 2008; Sherman and Sherman, 1983). Similarly, in *Anabaena* sp. PCC 7120, iron depletion causes PSI tetramer disintegration because PsaL, required for tetramer formation, competes with IsiA for binding to the PSI monomer. Increased IsiA production under iron limitation thus favors the formation of PSI monomers with IsiA in place of PsaL (Nagao et al., 2023). Long-term iron deficiency leads to the accumulation of unbound IsiA, which quenches excited Chl molecules. This causes dissipation of energy as heat and results in decreased Chl fluorescence. Excitation energy from Chl *a* is transferred to a cysteine residue in IsiA for thermal dissipation (Chen et al., 2017; Schoffman and Keren, 2019; Chen et al., 2021). Thus, IsiA protects cyanobacteria from the damaging effects of detached PBSs and Chl *a* molecules. Considering the function of IsiA protein, caution should be taken when growing cyanobacterial cultures on a large scale under natural light condition. This is because the combined effect of iron limitation and high light intensity can lead to unwanted thermal dissipation of excitation energy, which may reduce biomass production.

#### High-light-inducible protein (HliP)

HliPs are part of the LHC superfamily and were first identified in *Synechococcus elongatus* PCC 7942, which lacks OCP-based NPQ and utilizes Hlip for photoprotection under high light. Among various LHCs (Konert et al., 2023), HliPs are the most ancient, characterized by a single-helix structure with the ExxNxR Chl-binding motif, typical of LHCs (Komenda and Sobotka, 2016; Konert et al., 2023). These proteins share homology with one-helix proteins (OHPs) in higher plants and algae, which bind Chl *a/b* pigments (Konert et al., 2023). Ubiquitous in cyanobacteria, HliPs are involved in Chl biosynthesis, PSII repair, and photoprotection (Konert et al., 2023; Krynická et al., 2023). The expression of *hli* genes is induced by high light and oxidative stress. Also, the accumulation of HliPs is regulated by FtsH4 protease, which in turn ensures the proper biogenesis and assembly of PSII (Krynická et al., 2023). Furthermore, their interaction with Chl and Cars suggests a role in NPQ, which highlights their importance under high light in OCP-lacking cyanobacteria (Komenda and Sobotka, 2016; Konert et al., 2023). Information on the global importance of HliPs in cyanobacteria is limited, and therefore, further genomic, transcriptomics, proteomics, mutagenesis, and gene overexpression studies are needed to establish their importance across a wider range of organisms.

### State transition ensures balanced excitation of PSII and PSI

Fluctuating light can cause an imbalance in the excitation of PSII and PSI, leading to compromised photosynthesis and photooxidative stress (Calzadilla and Kirilovsky, 2020). This imbalance is resolved through state transitions, a reversible process where the peripheral antenna, or light-harvesting complex (LHC), of PSII migrates to PSI (Calzadilla and Kirilovsky, 2020). In higher plants, state transitions are controlled by thylakoid membrane-bound threonine kinase and phosphatase enzymes. These enzymes facilitate the phosphorylation and dephosphorylation of the light-harvesting complex (LHCII), respectively, which triggers its movement between the two PSs (Haldrup et al., 2001; Calzadilla and Kirilovsky, 2020). The signal for LHCII phosphorylation arises from the accumulation of a reduced PQ pool, a consequence of PSII overexcitation. This triggers the transition from state 1 to state 2, leading to the detachment of LHCII from PSII and its subsequent association with PSI. During state 2, PSI is preferentially excited, causing the PQ pool to become oxidized. This oxidation event activates the phosphatase, which then dephosphorylates LHCII, prompting its migration back from PSI to PSII, thus completing the transition from state 2 to state 1 (Calzadilla and Kirilovsky, 2020).

In cyanobacteria, PBSs mediate the distribution of excitation energy between PSII and PSI. The redox state of the PQ pool influences the electronic load on both PSs, triggering the reversible movement of PBSs between state 2 and state 1 (Calzadilla and Kirilovsky, 2020). State transitions can be studied using 77 K fluorescence spectrophotometry, where the relative change in PSII to PSI fluorescence intensity reflects the excitation energy distribution (Kaňa et al., 2012). A preferential transfer of excitation energy from PBSs to PSI results in a decrease in PSII fluorescence yield. Notably, Murata, 1969 first observed that prolonged illumination with blue or far-red light, preferentially absorbed by PSI, induces the state 2 to state 1 transition, characterized by a high PSII:PSI fluorescence ratio.

State transitions occur naturally during illumination but can also be induced by the non-photochemical reduction of the PQ pool through respiration in darkness or low light conditions (Chukhutsina et al., 2015). This can lead to inaccurate measurements of minimum fluorescence (F_0_) and maximum fluorescence (F_m_), resulting in an underestimation of the maximum quantum efficiency of PSII (F_v_/F_m_) due to the reduced PQ pool (Stirbet et al., 2019). Here, F_0_ represents the minimum Chl *a* fluorescence when the reaction center is open (oxidized PQ pool), while F_m_ represents the maximum fluorescence when the reaction center is closed (reduced PQ pool). To address this issue, dark-adapted cells are briefly illuminated with a weak beam of blue-green or far-red light (preferentially absorbed by the high Chl content in PSI) to oxidize PQH_2_ (Tiwari et al., 2024). This treatment is commonly employed to induce a state 1 transition, which helps in obtaining a more accurate F_0_ value in cyanobacteria (Stirbet et al., 2019).

### Respiratory cofactors protect cyanobacteria under high light

Respiratory cofactors are crucial in mitigating the damaging effects of high light, which imposes a combined burden of photons and electrons on the photosynthetic machinery. Located in close proximity to PSI, these cofactors protect the system by regulating electron transport (Liu, 2016). When the rate of carbon fixation lags behind electron production, NADPH accumulates in the cytosol. To counteract this, the NDH-1 enzyme, situated in the TM, increases the concentration of NADP^+^ in the cytosol and protons in the lumen (Lea-Smith et al., 2016). Additionally, the succinate dehydrogenase (SDH), also located in the TM, reduces the PQ pool by oxidizing succinate to fumarate (Lea-Smith et al., 2016). The thylakoidal respiratory electron transport chain also features terminal oxidases, Cyd and Cox. Under high-light conditions, these oxidases compete with PSI for electrons from PQH_2_ and either Pc or Cyt *c*_6_, respectively. This competition helps in preventing the formation of ROS by utilizing protons and oxygen to produce water (Ermakova et al., 2016).

### Protective role of flavodiiron proteins (FDPs) in minimizing ROS production

Flavoproteins (Flvs), also known as FDPs, are prevalent in anaerobic and some aerobic prokaryotes and protozoa (Allahverdiyeva et al., 2015). Despite their diverse classifications, all FDPs share two highly conserved structural domains. The N-terminal domain features a metallo-β-lactamase-like module containing a non-heme diiron center (Allahverdiyeva et al., 2015). This domain is specialized for the reduction of nitric oxide (NO) and O_2_, thereby preventing the intracellular accumulation of ROS. X-ray crystallographic studies of FDPs from various organisms have elucidated the electron transfer mechanism to O_2_ and/or NO within the enzyme’s active site (Allahverdiyeva et al., 2015). Connecting the N and C-terminal domains is an intermediate functional domain. The C-terminal domain adopts a flavodoxin-like fold and contains a flavin mononucleotide (FMN) moiety, exhibiting NADPH-flavin oxidoreductase activity (Allahverdiyeva et al., 2015).

FDPs can be classified based on the diverse extensions present in their C-terminal domains (Romão et al., 2016). Class A represents the largest group of the simplest FDPs, possessing only the FMN-binding domain described above. This class is commonly found in bacteria, archaea, and protozoa. Class B FDPs, found in enterobacteria, contain an Fe-S cluster in addition to the FMN-binding domain. Class C FDPs are obligately present in all oxygenic photoautotrophs and are characterized by an NAD(P)H:flavin oxidoreductase-like domain. Class D FDPs are restricted to protozoa and some bacteria and contain a flavin adenine dinucleotide (FAD) domain alongside an Fe-S cluster (Martins et al., 2019; Allahverdiyeva et al., 2015).

FDPs are encoded by *flv* genes, which exist in multiple copies within the cyanobacterial genome, forming small families of distinct FDPs. In oxygenic photoautotrophs, FDPs are categorized into two clusters: A and B (Allahverdiyeva et al., 2013). Cluster A includes Flv1 and Flv2, whereas Cluster B comprises Flv3 and Flv4. Members from these clusters form heterodimers, such as Flv1/Flv3 and Flv2/Flv4, to execute their functions (Romão et al., 2016; Santana-Sanchez et al., 2019).

Anaerobes exhibit high sensitivity to oxygen, making FDPs essential for their survival. In these organisms, FDPs reduce O_2_ to water, safeguarding cells from oxidative stress (He et al., 2025). Mehler (1951) and Mehler and Brown (1952) initially described the photoreduction of O_2_ to H_2_O_2_ by the photosynthetic electron transport chain in chloroplasts, a process known as the Mehler reaction. Subsequent research indicated that the primary product of this reaction is the superoxide anion. This anion is then converted to H_2_O_2_ by superoxide dismutase, which is further reduced to water by ascorbate peroxidase (Forti and Ehrenheim, 1993). In this cycle, electrons originate from the splitting of water at the PSII side and are ultimately transferred to O_2_ via PSI (Asada, 2000). Consequently, this process is also termed pseudocyclic electron flow or the water–water cycle. The pseudocyclic electron flow or water–water cycle serves two critical functions: first, it prevents saturation of the Z-scheme by diverting excess electrons from PSI to O_2_ when CO_2_ assimilation is limited. Second, it contributes to the generation of a proton gradient across the TM, thereby inducing NPQ and ATP synthesis (Foyer and Noctor, 2000; Allahverdiyeva et al., 2013).

The Flv1/Flv3 heterodimer also plays a role in state transitions by facilitating the oxidation of the PQ pool when carbon fixation by RuBisCO is low (Bersanini et al., 2014; He et al., 2025). Furthermore, under fluctuating light conditions, this heterodimer maintains the redox balance within the electron transport chain, protecting cells from oxidative damage that can arise from an imbalance in electron flow between PSII and PSI, leading to a decrease in photosynthetic rate (Allahverdiyeva et al., 2015; He et al., 2025). Studies on knockout and overexpression strains of *flv2/flv4* suggest the existence of an alternative electron transport pathway to an unidentified acceptor near the Q_B_ site (Santana-Sanchez et al., 2019). The Flv2/Flv4 heterodimer stabilizes forward electron transfer in PSII, thereby enhancing charge separation and preventing the formation of singlet oxygen, which can occur through energy transfer from excited Chl to O_2_ (Bersanini et al., 2014). The expression of *flv2* and *flv4* genes is elevated in the absence of OCP, and their protein products contribute to the stabilization of the PSII dimer and its association with PBSs (Bersanini et al., 2014). In conclusion, the FDPs represent a critical and highly-evolved protective system in cyanobacteria. Their ability to manage electron flow and dissipate excess energy plays fundamental role in maintaining redox homeostasis and preventing photodamage under fluctuating light conditions. Therefore, continued study of these proteins is essential for a complete understanding of their complex roles and leverages their function for biotechnological applications.

## Other photoprotective mechanisms in cyanobacteria

As a first line of defense against high-energy PAR and UVR, cyanobacterial cells employ physical barriers. These include the production of a mucilaginous sheath and photoprotective compounds such as mycosporine-like amino acids (MAAs) and scytonemins (Singh et al., 2010; Singh et al., 2023b). Filamentous forms can also create amorphous silica matrices, form mats, and undergo morphological changes to reduce their surface area, providing further protection against intense PAR and UVR (Singh et al., 2010; Singh and Montgomery, 2011). Additionally, these organisms exhibit phototactic and photophobic responses to adjust their position relative to light intensity (Gupta et al., 2023). Beyond these strategies, cyanobacteria possess a range of enzymatic (superoxide dismutase, catalase, glutathione peroxidase, ascorbate peroxidase, and monodehydroascorbate reductase) and non-enzymatic (ascorbate, α-tocopherol, and reduced glutathione) mechanisms to keep ROS at tolerable levels (Latifi et al., 2009). Furthermore, if genomic DNA is directly damaged by UVR absorption or indirectly by ROS production, cyanobacteria can utilize various repair mechanisms to prevent mutations caused by extreme light environments (Pathak et al., 2019; Singh et al., 2023a).

## Biotechnological implications and future perspectives

In summary, cyanobacteria, ancient and metabolically versatile prokaryotes, have profoundly influenced on Earth’s environment and remain ecologically significant. Their remarkable oxygenic photosynthesis not only paved the way for aerobic life and the ozone layer but also holds immense promise for sustainable biotechnological applications. However, realizing this potential depends on the resilience of their photosynthetic machinery under fluctuating light environment. Cyanobacteria have evolved photoprotective and photoacclimatory mechanisms, including energy dissipation pathways, antioxidative systems, and chromatic acclimation, to optimize light harvesting and maintain photosynthetic efficiency across diverse and dynamic environments. Understanding these intricate light responses in detail is crucial for both comprehending their ecological success and effectively harnessing their metabolic capabilities for a sustainable future via biomanufacturing.

The extensive adaptive and acclimative strategies of cyanobacteria under fluctuating light conditions make them more suitable than algae and higher plants for open or closed cultivation systems, where light fluctuation is a major concern. Due to the dense cell population in these systems, light intensity is limited and enriched in green-blue wavelengths. This reduces the quantum yield of photosynthesis in algae and higher plants because they lack the photoacclimation strategies found in cyanobacteria. Therefore, the genome bank of cyanobacteria presents an enormous opportunity to engineer photoautotrophs of biotechnological importance for better growth and performance in large-scale cultivation systems. However, the fact that no single cyanobacterial strain possesses the complete repertoire of light stress mitigation mechanisms presents a compelling avenue for future research and bioengineering. Expanding the “umbrella of tolerance” within a single strain by integrating a comprehensive suite of light-adaptive features into a single genome holds significant promise.

For instance, engineering strains to robustly manage excitation pressure through the enhanced expression and coordinated action of proteins like OCP, HliPs, and IsiA could lead to substantial improvements in photochemical efficiency. This would not only conserve cellular energy otherwise invested in mitigating electronic load and repairing cellular machineries under high light but could also intrinsically regulate the rate and content of ROS production. However, constitutive expression of such protective mechanisms can compromise the productivity under light limiting conditions due to loss of excess energy. Considering the growing economic and pharmaceutical significance of cyanobacteria for humankind, the development of such “super-resilient” strains with minimized ROS production presents a highly attractive prospect for various industrial applications. Future efforts should therefore prioritize the genetic engineering and synthetic biology approaches necessary to consolidate these diverse light-acclimation traits within a single, high-performing cyanobacterial strain. The research on how salt stress affects stability of PSI (in trimer or tetramer) and interaction of OCP with PBS and PSs is still very limited This could unlock new frontiers in sustainable biofuel production, carbon capture, bioremediation, and the synthesis of valuable bioactive compounds (biomanufacturing), ultimately leveraging the remarkable photosynthetic capabilities of cyanobacteria for achieving sustainable developmental goals.
